# Neurocognitive function in bipolar disorder: a comparison between bipolar I and II disorder and matched controls

**DOI:** 10.1186/1471-244X-13-165

**Published:** 2013-06-07

**Authors:** Erik Pålsson, Clara Figueras, Anette GM Johansson, Carl-Johan Ekman, Björn Hultman, Josefin Östlind, Mikael Landén

**Affiliations:** 1 Institute of Neuroscience and Physiology, University of Gothenburg, Gothenburg, Sweden; 2 Department of Clinical Neuroscience, Karolinska Institutet, Stockholm, Sweden; 3 Department of Medical Epidemiology and Biostatistics, Karolinska Institutet, Stockholm, Sweden

## Abstract

**Background:**

Cognitive deficits have been documented in patients with bipolar disorder. Further, it has been suggested that the degree and type of cognitive impairment differ between bipolar I and bipolar II disorder, but data is conflicting and remains inconclusive. This study aimed to clarify the suggested differences in cognitive impairment between patients with bipolar I and II disorder in a relatively large, clinically stable sample while controlling for potential confounders.

**Methods:**

67 patients with bipolar I disorder, 43 with bipolar II disorder, and 86 randomly selected population-based healthy controls were compared. A number of neuropsychological tests were administered, assessing verbal and visual memory and executive functions. Patients were in a stable phase during testing.

**Results:**

Patients with bipolar type I and type II were cognitively impaired compared to healthy controls, but there were no statistically significant differences between the two subtypes. The strongest predictor of cognitive impairment within the patient group was current antipsychotic treatment.

**Conclusions:**

The present study suggests that the type and degree of cognitive dysfunction is similar in bipolar I and II patients. Notably, treatment with antipsychotics - but not a history of psychosis - was associated with more severe cognitive impairment. Given that patients with bipolar I disorder are more likely to be on antipsychotic drugs, this might explain why some previous studies have found that patients with type I bipolar disorder are more cognitively impaired than those with type II.

## Background

Decreased concentration and attention are common complaints among bipolar patients. Such cognitive impairment can be objectively measured with neuropsychological tests [1]. Growing evidence from the last two decades has shown that not only is cognitive dysfunction evident during depressive or manic episodes, but the impairment also persists during the euthymic phases of the illness [2-4]. The cognitive domains most consistently found to be impaired are executive functioning, attention, and verbal memory [5-7].

Although neurocognitive deficits have accordingly been documented in bipolar I disorder, a recently published systematic review [8] concluded that studies of patients with bipolar disorder type II are inconsistent. Elucidating whether cognitive dysfunction is a general trait across bipolar subtypes or differs between type I and II not only has implications for rehabilitation programs, but would also shed light on the validity of classifying bipolar subtypes and hence pathophysiological research. It has for example been suggested that the bipolar subtypes I and II are qualitatively different entities with different underlying pathophysiology [9]. If this is true, then differing pathophysiology might be mirrored by different patterns of cognitive impairment, which might serve as a more reliable phenotype than categorical diagnoses [10]. In support of this notion, one study found that cognitive dysfunction in bipolar I patients differed not only in magnitude, but also in pattern compared with bipolar II patients [11]. Whereas both subtypes showed deficits in working memory, verbal fluency, and interference control compared to healthy controls, bipolar I patients were more severely impaired overall and showed deficits in verbal learning and verbal memory that were not seen in bipolar II patients. At odds with this notion, however, a second study found quantitative, but no qualitative neurocognitive differences between the bipolar subtypes [12]. In the latter study, bipolar II patients showed an intermediate level of performance in verbal memory and executive functions between bipolar I patients and healthy controls, agreeing with a view where bipolar type I and II represent varying degrees of disease severity on a continuum. Finally, a third study found no differences in neurocognitive function between bipolar type I and type II patients [13]. Here, both bipolar patients type I and type II performed worse than healthy controls in most cognitive domains with the largest effect size in psychomotor speed, working memory, verbal learning, and executive functions. Hence, this last study suggests that cognitive function cannot be used to discriminate between bipolar disorder type I and type II. Apart from these relatively large studies, several smaller studies show worse cognitive performance in bipolar type I compared to bipolar type II patients [14,15]. However, a recent meta-study also concluded that, with the exception of semantic fluency and visual memory, the cognitive deficits in bipolar II patients are as severe as in bipolar I patients [16].

Given the inconsistency of available data regarding potential cognitive differences between the two bipolar subtypes I and II, the aim of the present study was to compare the cognitive performance between bipolar disorder type I and bipolar disorder type II patients in a relatively large and clinically stable study sample. An extensive cognitive test battery was used and all patients had a validated diagnosis of bipolar disorder. Confounders, such as ongoing medication, residual mood symptoms or a history of psychosis, were controlled for. For reference, randomly selected population-based age and sex matched healthy controls were recruited. The use of population-based controls is important since control groups recruited among hospital staff, students, or through advertisements confers substantial risk for sample bias.

## Methods

The present study is part of the St. Göran bipolar project, which provides assessment, treatment, and follow-up of patients with bipolar disorder within the Northern Stockholm Mental Health Service and also serves as a basis for research into bipolar disorder. The methodology has previously been outlined in detail [17-19]. A total of 67 patients with bipolar disorder type I, 43 patients with bipolar disorder type II and 86 controls were included in this study.

### Clinical assessments

Patients were assessed by a psychiatrist or resident in psychiatry using the Affective Disorders Evaluation (ADE), which is a standardized protocol adapted from the Systematic Treatment Enhancement Program of Bipolar Disorder (STEP-BD) [20]. The ADE guides the interviewer through a systematic assessment of the patient's current and past mental state, and provides a diagnosis according to DSM-IV criteria. The number of lifetime affective episodes and their characteristics are documented. Other modules assess alcohol and drug misuse, violent behavior, childhood history, family history, treatment history, reproductive history, and somatic illnesses. Interpersonal violence is defined as a violent act or serious physical threat to another person. Suicide attempt is defined as a deliberate and serious self- injury, including intoxication with medication.

The final diagnosis was established using LEAD (Longitudinal observation by Experts using All Data) [21] and confirmed by a consensus panel of two to four experienced clinicians. Inclusion criteria for this substudy were bipolar I or II diagnosis. Disease severity was assessed using the clinician rated Global Assessment of Function (GAF) and Clinical Global Impression (CGI) scales [22,23]. Mood stability was determined by the treating physician's overall diagnostic judgment. The Montgomery-Åsberg depression rating scale (MADRS) and Young Mania rating scale (YMRS) were administered by the neuropsychologist at the first cognitive testing session to assess residual mood symptoms. Patients with MADRS > 14 or YMRS > 14 were not included in this study.

### Control group

Age- and sex-matched persons for each enrolled patient were selected randomly from the national population register by Statistics Sweden (http://www.scb.se) and contacted by letter. Persons who were interested in participating in the study contacted the study team that conducted a preliminary telephone screening to exclude severe mental health and neurological issues as well as substance abuse. Eligible individuals were scheduled for a one-day comprehensive assessment. Exclusion criteria for controls were: 1) any on-going psychiatric or neurological disorder; 2) current treatment with any psychotropic drugs; 3) past history of bipolar disorder, schizophrenia, recurrent depression or other psychiatric disorder leading to extended sick leave; 4) a first-degree relative with schizophrenia or bipolar disorder; 5) subjects presenting conditions that precluded magnetic resonance imaging of the brain (e.g., metal implants, shrapnel, and certain heart operations). The latter exclusion criterion was due to a planned brain MRI scan for another part of the St. Göran bipolar project. Two of the recruited controls were excluded due to one case of dementia diagnosis and one case of alcohol addiction that was revealed at the examination.

During the one-day assessment, a psychiatrist interviewed eligible control subjects in a semi-structured manner utilizing relevant sections of the ADE and the Mini International Neuropsychiatric Interview (MINI) [24] as a screen for past and present psychiatric disorders. This included a screen for bipolar illness as well as questions about socio-economic status, use of alcohol and psychoactive substances, history of violent, criminal or suicidal behavior (defined as deliberate and serious self-injury including intoxication with medication). Furthermore, childhood history, family history of psychiatric disorders in first and second-degree relatives, treatment history, reproductive history and somatic illnesses were reviewed. The Mini International Neuropsychiatric Interview (MINI) [24] was used to screen for other psychiatric disorders than bipolar illness. The GAF was used to assess axis V. Included subjects also completed three self-report questionnaires amongst which were: the Alcohol Use Disorders Identification Test (AUDIT) [25] and the Drug Use Disorders Identification Test (DUDIT) [26]. Finally, all subjects underwent neuropsychological testing by senior clinical psychology students under supervision of the same experienced clinical psychologist that supervised and conducted testing in the patient group.

### Neuropsychological measures

Study participants were tested using a number of neuropsychological tests. All tests were administered according to standard instructions. Patients were tested during a stable phase of the disorder. Administering all tests usually required two sessions with patients, whereas all controls were tested during one single session.

The instruments included:

a) Five tests from the Delis-Kaplan Executive function system D-KEFS [27]. The D-KEFS is a standardized set of tests designed to assess executive functions including flexibility of thinking, inhibition, problem solving, planning, impulse control, concept formation, abstract thinking, and creativity. It consists of nine game-like tests that can be administrated as a complete battery or as individual subtests. Here, we chose five out of nine tests: Trail making test, Design fluency test, Verbal fluency test, Color-word interference test, and Tower test. The D-KEFS Trail making test consists of a visual cancellation task and a series of connect-the-circle tasks. The primary executive-function task is condition 4 (number-letter switching), which assesses flexibility of thinking on a visual-motor sequencing task. The other four conditions of this test allow the examiner to evaluate other processes necessary for performing the task, including visual scanning, number sequencing, letter sequencing, and motor speed. The D-KEFS Verbal fluency test measures the ability to generate words fluently in a difficult phonemic format (letter fluency), from overlearned concepts (category fluency), and simultaneously shifting between overlearned concepts (category switching). The test gives information about language skills and verbal processing ability, as well as problem solving. The D-KEFS Design fluency test measures the ability to draw as many different designs as possible in 60 seconds. It comprises 3 conditions. Condition 1 provides a basic test of design fluency, condition 2 measures both design fluency and response inhibition, and condition 3 measures design fluency and cognitive flexibility. Basic skills involved in this test are visual attention, motor speed, visual-perceptual skills, and constructional skills. The executive functions required include visual/ spatial initiation of problem-solving behavior, fluency, creativity, simultaneous processing and inhibiting capacity. D-KEFS Color-word interference test corresponds to the Stroop color word test and primarily measures the ability to inhibit an overlearned verbal response, in this case reading the printed words, and to generate the conflicting response of naming the dissonant ink colors in which the words are printed. This is also a test of cognitive flexibility. The D-KEFS Tower test assesses planning and spatial problem solving abilities such as the ability to inhibit perseverative and impulsive responses. Visual attention and visual-spatial ability are fundamental skills for this task.

b) Claeson-Dahl learning and memory test was designed to evaluate episodic memory. The test has three dimensions. Initially the subject is asked to learn through hearing a list of ten words. The retention dimension involves remembering as many words as possible from the original list and to remember what order the words was originally read in. Finally, in the recognition dimension, the test person is asked to recognize the words from the list among similar distractors.

c) In the Rey complex figure test, the test person is asked to reproduce a complicated drawing, initially by copying and by recall. The test draws on cognitive domains such as memory, visuospatial ability, attention processes, planning ability, and incidental learning.

### Statistical analysis

Group-wise comparisons were done using ANOVA for continuous variables and chi-square tests for dichotomous variables. Bonferroni post hoc tests were used where relevant. Associations between cognitive performance and other variables were tested using Pearson correlation or forward selection stepwise linear regression modelling. Two-tailed levels of significance were used and p < 0.05 was considered statistically significant.

### Ethics

All participating patients and control subjects consented orally and in writing to participate in the study. The project was approved by the Stockholm Regional Ethical Review Board and conducted in accordance with the latest version of the Helsinki Protocol. The healthy subjects received remuneration for their participation.

## Results

### Demographics

#### Subjects

The most frequent pharmacological treatments and other characteristics of the three groups are shown in Table 1. The three groups (bipolar I, bipolar II, and healthy controls) did not differ with respect to gender, age, or educational level (Table 1). The two patient groups did not differ in age at onset. Patients with bipolar disorder type I showed a significantly higher occurrence of psychotic symptoms, and were more often treated with lithium and antipsychotics than type II. The fact that some bipolar II patients had a history of psychotic features is explained by psychotic symptoms during depressive episodes. Type II patients had more depressive episodes.

**Table 1:** Demographic and clinical characteristics of the study sample

	**Control (N = 86)**	**Bipolar I (N = 67)**	**Bipolar II (N = 43)**	**ANOVA**		**P**
				**F**	**χ**^**2**^	
Age, years: mean (SD)	38.1 (14.5)	38.3 (13.5)	35.7 (12.0)	0.562		0.57
Gender (male / female)	39 / 47	32 / 35	16 / 27		1.24	0.54
Educational level,1-5: mean (SD)^a^	4.1	3.8	3.8	1.08		0.34
WAIS_information_ subtest	13,3 (2,3)	13,2 (2,3)	13,6 (2,2)	0,38		0,69
Age of onset: mean (SD)		20.6 (8.4)	19.1 (8.5)	0.88		0.35
Prior psychotic symptoms: N (%)		52 (78)	7 (17)		39.6	< 0.001
Suicide attempt: N (%)		26 (39)	16 (38)		0.06	0.94
Interpersonal violence: N (%)		20 (30)	10 (23)		0.57	0.45
Anxiety disorder		19 (28)	24 (56)		7.96	0.005
ADHD		7 (11)^c^	10 (23)		3.04	0.081
MADRS: mean (SD)		3.9 (3.5)	3.6 (3.6)	0.17		0.68
YMRS: mean (SD)		1.6 (2.7)	2.2 (2.2)	1.2		0.28
Lifetime number of manic episodes: mean (SD)		3 (2.9)	-			
Lifetime number of depressive episodes: mean (SD)		13.3 (13.2)	28.5 (31.0)	12.4		0.001
CGI: mean (SD)		4.9 (0.9)	4.1 (0.7)	23.2		< 0.001
Pharmacological treatment, N (%)						
Lithium		49 (73)	21 (49)		6.7	0.01
Anticonvulsant		15 (22)	8 (19)		0.22	0.63
Antidepressant		18 (27)	19 (44)		3.5	0.061
Antipsychotic		22 (33)	6 (14)		4.9	0.027
Combination therapy^b^		34 (51)	17 (40)		1.32	0.25

^a^1 = less than 9 years, 2 = 9 years, 3 =12 years, 4 = 13–15 years and 5 = more than 15 years of education.

^b^Combination of 2 or more psychotropic medications.

^c^Missing data for 2 patients.

### All bipolar patients versus controls

The results of the neuropsychological test scores are shown in Table 2. Overall, patients performed significantly worse than controls on all trials in the Verbal fluency task, aspects of the Design fluency, Tower, Rey complex figure and Trail making tests, but not on the Claeson-Dahl memory task. The largest effect sizes were observed for the number sequencing and number-letter switching conditions of the Trail making test, time first move and time per move in the Tower test, semantic and set-shifting trials of the Verbal fluency test and set-shifting trial of the Design fluency test.

**Table 2:** Performance on neuropsychological tests for patients with bipolar disorder type I, type II and healthy controls with data presented as mean (SD) scores

	**Bipolar I (BPI)**	**Bipolar II (BPII)**	**Control (C)**	**ANOVA**	**P**	**Bonferroni post hoc test**	**Cohen’s D**		
	**Mean (SD)**	**Mean (SD)**	**Mean (SD)**				**BPI vs C**	**BPII vs C**	**BPI vs BPII**
Claeson-Dahl^1^	N = 57	N = 42	N = 84						
Learning	46.9 (11.0)	47.2 (12.1)	49.3 (10.3)	1.04	0.35				
Retention	48.0(12.8)	44.0 (12.5)	48.0 (10.4)	1.97	0.14				
Recognition	9.6 (0.8)	9.7 (0.7)	9.8 (0.6)	1.19	0.31				
Colour-Word^2^	N = 60	N = 40	N = 82						
Colours	8.7 (3.5)	9.1 (2.2)	9.8 (2.3)	2.66	0.072				
Colour names	9.7 (2.8)	10.5 (1.9)	10.1 (2.5)	1.28	0.28				
Inhibition	9.6 (3.7)	9.9 (2.6)	11.7 (2.5)	9.70	<0.001	BPI, BPII < C	0.54	0.55	0.09
Inhibition and set-shifting	9.6 (3.4)	10.1 (2.7)	11.3 (2.6)	6.73	0.002	BPI < C	0.56	0.45	0.16
Design Fluency^2^	N = 65	N = 40	N = 84						
Draw designs	11.1 (3.2)	11.3 (2.4)	11.7 (2.7)	0.97	0.38				
Connect the dots	11.1 (3.0)	11.2 (3.0)	11.5 (2.3)	0.48	0.62				
Set-shifting	10.9 (2.7)	11.5 (2.7)	13.1 (2.7)	12.87	<0.001	BPI, BPII < C	0.81	0.59	0.22
Rey Complex Figure ^3^	N = 60	N = 42	N = 80						
Copy score	33.6 (2.7)	32.7 (3.1)	32.7 (3.1)	0.28	0.76				
Time to draw figure	221.8 (140.3)	199.6 (101.3)	159.7 (71.6)	6.35	0.002	BPI > C	0.56	0.45	0.18
Immediate recall	41.8 (14.0)	44.4 (15.0)	48.4 (12.9)	4.94	0.008	BPI < C	0.49	0.29	0.18
30 minute recall	43.4 (14.8)	43.0 (14.2)	46.9 (13.2)	2.24	0.11				
Recognition	43.4 (11.5)	44.5 (13.5)	50.2 (11.3)	6.84	0.001	BPI, BPII < C	0.60	0.46	0.08
Tower^4^	N = 58	N = 35	N = 56						
Total score	10.5 (3.6)	11.1 (3.3)	11.9 (2.4)	3.22	0.043	BPI < C	0.46	0.28	0.17
Rule violations	1.3 (3.0)	1.2 (4.1)	0.3 (0.7)	2.44	0.091				
Time first move	9.0 (3.5)	8.5 (2.3)	10.6 (2.8)	7.01	0.001	BPI, BPII < C	0.50	0.82	−0.17
Time per move	7.6 (3.6)	8.5 (2.7)	10.3 (2.3)	13.36	<0.001	BPI, BPII < C	0.89	0.72	0.28
Move accuracy	11.4 (3.3)	10.6 (2.6)	10.3 (2.0)	2.33	0.10				
TMT^2^	N = 65	N = 41	N = 85						
Visual Scanning	10.5 (2.9)	11.1 (1.8)	10.8 (2.3)	0.85	0.43				
Number Sequencing	8.8 (3.6)	10.0 (2.8)	11.5 (2.4)	15.18	<0.001	BP, BPII < C	0.88	0.58	0.37
Letter Sequencing	9.3 (3.1)	9.8 (2.8)	11.1 (2.7)	7.72	0.001	BPI < C	0.62	0.47	0.17
Number-Letter Switching	9.1 (3.0)	9.4 (2.9)	11.6 (1.6)	22.99	<0.001	BPI, BPII < C	1.04	0.94	0.10
Motor Speed	11.4 (2.2)	11.6 (2.4)	11.8 (1.6)	0.92	0.40				
Verbal Fluency^2^	N = 65	N = 43	N = 83						
Phonetic	12.0 (4.6)	12.4 (4.0)	13.7 (3.3)	3.88	0.022	BPI < C	0.42	0.35	0.09
Semantic	12.3 (4.4)	12.4 (4.0)	16.0 (3.2)	20.72	<0.001	BPI, BPII < C	0.96	0.99	0.02
Set-shifting	10.9 (3.6)	11.5 (3.1)	13.7 (3.4)	12.98	<0.001	BPI, BPII < C	0.91	0.68	0.18

^1^Mean scores are t-scores for learning and retention and raw scores for recognition.

^2^Mean scores are demographics corrected standard scores.

^3^Mean scores are raw scores for copy score and time to draw figure and t-scores for recall and recognition variables.

^4^Mean scores are demographics corrected standard scores except rule violations, which is a raw score.

### Bipolar disorder type I versus type II

There were no statistically significant differences between bipolar I and bipolar II patients. Bipolar I patients did show an overall tendency towards more impairment as demonstrated by the post-hoc tests and magnitude of effect sizes.

### Potential confounders

To reduce the number of comparisons, further analysis was restricted to those neuropsychological test variables where a significant difference between patients groups and the control groups was demonstrated. Since no significant differences were shown between patients with type I and type II bipolar disorder, the patient samples were pooled for the remaining analyses. Treatments with psychotropic drugs, residual mood symptoms and a history of psychosis have all previously been shown to influence cognitive performance. Linear regression analysis using a step-wise procedure with the most prevalent drug classes in the patient group, history of psychotic symptoms and MADRS and YMRS scores as independent and neuropsychological test scores as dependent variables were performed. Anxiety disorder or ADHD diagnosis were also included to control for co-morbidity.

There were no significant associations between cognitive test performance and treatment with lithium and anticonvulsants. A single association was found between treatment with antidepressants and the letter sequencing part of the Trail making test. Conversely, treatment with antipsychotics was associated with worse performance on the time to draw parameter of the Rey complex figure test, number sequencing, letter sequencing and number-letter switching conditions of the Trail making test and all trials of the Verbal fluency test. Examining the patient group, there were no significant associations between cognitive test performance and MADRS score, history of psychosis or ADHD diagnosis. Anxiety disorder diagnosis was associated with worse performance on the phonetic part of the Verbal fluency test. Current mania symptoms were associated with worse performance on the time to draw parameter of the Rey complex figure test, the inhibition trial of the color-word test and the phonetic and semantic parts of the Verbal fluency test. The results of this analysis are shown in Table 3.

**Table 3:** Performance on neuropsychological tests and patient data

**Test**	**Predictor variable**	***β***	**t**	**df**	**p**
Colour-Word	*Inhibition*				
	YMRS	−0.22	−2.06	1	0.042
	*Inhibition and set-shifting*				
	Antipsychotics	−0.25	−2.42	1	0.017
Rey complex figure	*Time to draw figure*^*1*^				
	Antipsychotics	0.29	2.92	1	0.004
	YMRS	0.21	2.12	1	0.037
Tower test	*Total score*				
	Antipsychotics	−0.24	−2.25	1	0.027
TMT	*Number Sequencing*				
	Antipsychotics	−0.30	−2.90	1	0.005
	*Letter Sequencing*				
	Antipsychotics	−0.45	−4.50	1	<0.001
	Antidepressants	−0.25	−2.50	1	0.014
	*Number-Letter Switching*				
	Antipsychotics	−0.23	−2.18	1	0.032
Verbal fluency	*Phonetic*				
	Anxiety disorder	−0.27	−2.85	1	0.005
	Antipsychotics	−0.27	−2.88	1	0.005
	YMRS	−0.24	−2.49	1	0.015
	*Semantic*				
	YMRS	−0.29	−2.90	1	0.005
	Antipsychotics	−0.26	−2.62	1	0.010
	*Set-shifting*				
	Antipsychotics	−0.23	−2.20	1	0.030

Forward selection step-wise linear regression analysis of the each test variable was conducted using *MADRS*, *YMRS*, subdiagnosis, history of psychosis, anxiety disorder and *ADHD* co-morbidity and use or non-use of each medication group as independent variables. Only significant predictors for each test variable are listed in the table. ^1^Variable was log-transformed prior to analysis.

## Discussion

There are three main findings in the present study. First, the study confirmed previous findings that clinically stable patients with bipolar disorder showed impairment in inhibition, in set-shifting, in verbal fluency and in visual recall compared to healthy age-matched controls. Bipolar patients also gave more rapid responses during the Tower test and showed impulsivity on the Color-word test, yet were slower in drawing the Rey figure. Second, there were no major differences between the type I and type II subtypes of bipolar disorder. Third, treatment with antipsychotic drugs was associated with worse performance in verbal fluency, in inhibition, in set-shifting and a longer time to draw the Rey figure.

The patient group as a whole thus performed worse than controls on all the included tests from the D-KEFS battery. These tests tap different aspects of executive function and hence confirm previously observed deficits in several aspects of executive function including response inhibition, attention, working memory, cognitive flexibility, and spatial planning [3]. Further, patients performed worse on both the immediate recall and recognition parts of the Rey complex figure test. This was less apparent in the Claeson-Dahl test, which indicated reduced performance on visual but not verbal memory tasks. However, this finding may be confounded by differences in test sensitivity and the Claeson-Dahl test may not be a demanding enough test to detect verbal learning and memory deficits in the present patient sample. Other tests, such as the California verbal learning task may provide more sensitive alternatives [28].

Pertaining to the putative differences between bipolar I and II disorder, we observed no statistically significant differences. This is in line with the study by Dittmann et al. [13] and a recent study by Xu et al. [29], but conflicts the results by Torrent et al. [12] and Simonsen et al. [11]. One possible explanation for these conflicting findings is that the results are confounded by antipsychotic medication. In the present study, antipsychotic medication was coupled to significantly lower cognitive performance. This corroborates previous studies that have demonstrated that antipsychotics may have adverse effects on cognitive function in patients with bipolar disorder [30-32]. Given that bipolar I patients are more frequently treated with antipsychotics than bipolar II patients, this may be part of the explanation for previous observations of greater cognitive impairment in type I as compared to type II bipolar disorder.

A number of studies have linked psychotic features to more chronic and severe presentations of bipolar disorder [33]. Since impaired neurocognitive function has been linked to poor functional outcome, psychotic features and cognitive dysfunction may be connected. A recent study comparing psychotic and non-psychotic patients with schizophrenia, schizoaffective disorder, and bipolar disorder found that a history of psychosis was a stronger predictor than diagnostic group of neuropsychological test performance [34]. However, due to an almost complete overlap between history of psychosis and treatment with antipsychotic medication in that study, it was not possible to rule out an effect of antipsychotic drugs in that study.

Importantly and at odds with previous findings, we did not observe any association between previous psychotic symptoms and cognitive performance. In the present study, the overlap between history of psychosis and treatment with antipsychotic medication was only partial and could therefore be statistically controlled for. Indeed, our findings suggest that current treatment with antipsychotic medication predict neurocognitive test performance better than history of psychotic symptoms. Of interest is also the fact that the doses of antipsychotics in our study were low to moderate (data not shown), making this association all the more clinically important when deciding on treatment.

### Strengths and limitations

A particular strength of the present study is the population-based control sample. Comparisons between healthy controls and patients may be biased if the controls are unrepresentative of the general population. The use of a population-based control sample from the same catchment area indicates that the observed differences are unlikely to be the result of sampling bias, but reflect actual differences in neurocognitive function between patients and controls. Further, the meticulous diagnostic assessment ensures that the division in bipolar I and II disorder is as accurate as possible. However, it should be noted that the diagnostic procedure used here as not been analysed for reliability estimates which somewhat limits the conclusions that can be drawn regarding diagnostic accuracy. The sample is sufficiently large to avoid type II errors [8]. Finally, the present study used an extensive test battery providing a broad neurocognitive profiling.

With regards to the previous studies on cognitive function in bipolar II disorder our patient sample had a relatively young age of onset, 20 years versus approximately 28–30 years [11-13]. To a lesser extent this was also true for the bipolar I group with a mean age of onset of 19 years versus 24–28 years in previous studies. However, the mean age at first episode in our patient group was comparable to previously published demographic data [35]. Earlier age of onset has been linked to a more severe form of the disorder [36,37], although a late onset has been linked to a more pronounced cognitive deficit [38].

The cut-off for present mood symptoms used in this study was less strict than previous studies and lingering mood symptoms may influence cognitive test performance [39]. However, the mean scores for MADRS and YMRS were well below a more strict definition of euthymia [40] and the present study failed to demonstrate meaningful correlations between residual depressive symptoms and cognitive function. For residual manic symptoms, we observed a negative correlation between performance on the Verbal fluency test and YMRS score. Previous studies on neurocognitive function bipolar II disorder have found small [12] or no [11,13] effects of subsyndromal depressive and manic symptoms on cognitive function.

Our findings regarding the effects of current medication may be confounded by the fact that most patients are treated with more than one class of psychotropic medication. However, our analysis of combination therapy did not indicate any such effects. In addition, medication may obscure the influence of other factors, e.g., psychotic symptoms that may otherwise have been correlated with cognitive test scores. The fact that our study was cross-sectional also limits the conclusions that can be drawn regarding the relationship between cognitive function and clinical variables or treatment. Future longitudinal studies on the present patient sample may provide valuable information on the progression and significance of neurocognitive dysfunctions in the clinical setting.

## Conclusions

In conclusion, we found similar types and degrees of cognitive dysfunction in bipolar I and bipolar II patients as compared to healthy controls. Overall, the magnitude and cognitive domains affected are in agreement with previously published work on euthymic bipolar patients. We also found a greater degree of impairment in patients treated with antipsychotics, but no correlation between history of psychosis and cognitive function.

## Competing interests

The authors declare that they have no competing interests.

## Authors’ contributions

Authors ML, AJ, C-JE and BH designed the study and wrote the protocol. Authors EP and CF managed the literature searches and analyses. Author EP undertook the statistical analysis, and authors EP and CF wrote the first draft of the manuscript. All authors contributed to and have approved the final manuscript.

## Pre-publication history

The pre-publication history for this paper can be accessed here:

http://www.biomedcentral.com/1471-244X/13/165/prepub
